# Psychometric properties of the Persian social media intrusion questionnaire

**DOI:** 10.3389/fpsyg.2023.1084075

**Published:** 2023-02-09

**Authors:** Zahra Abedi, Ahmad Ashouri, Abbas Ramezani Farani, Hojjatollah Farahani

**Affiliations:** ^1^Department of Clinical Psychology, School of Behavioral Sciences and Mental Health (Tehran Institute of Psychiatry), Iran University of Medical Sciences, Tehran, Iran; ^2^Department of Psychology, Tarbiat Modares University, Tehran, Iran

**Keywords:** online social media, social networks, FIQ, SMIQ, confirmatory factor analysis

## Abstract

In recent years, social media use has dramatically increased worldwide, which has raised many concerns concerning the excessive use of social media. In this regard, Facebook Intrusion Questionnaire (FIQ) was developed to assess the level of addiction to Facebook. In this study, we first modified the FIQ items to cover all social media besides Facebook and labeled it the measure as Social Media Intrusion Questionnaire (SMIQ). Then, we examined its factor structure, reliability, and validity with 374 participants (M age = 25.91; *SD* = 5.81; 69.80% females) from the Iranian community sample. Confirmatory factor analysis supported the originally proposed uni-factor model, which was also invariant across gender groups. The SMIQ score demonstrated acceptable internal consistency (*α* = 0.85) and yielded expected associations with external correlates (e.g., cell-phone based addiction to social media, depression, and low self-esteem), supporting the measure’s convergent and divergent validity. Overall, our findings indicated that Persian SMIQ enjoys sound psychometric properties.

## Introduction

In recent decades, technological developments have significantly elevated the role of the Internet in our lives (e.g., Elhami Athar and Azamian Jazi, 2022). The Internet and social network sites (SNS) have provided an online communication medium as a popular way of creating and maintaining relationships (Elphinston and Noller, 2011). Therefore, it is likely that individuals spend excessive amounts of time on social media and develop a strong attachment to it. Studies have shown that the excessive use of social media is associated with adverse outcomes such as poor academic performance (e.g., Al-Yafi et al., 2018), social anxiety (e.g., Atroszko et al., 2018), depression (e.g., Satici, 2019), low life satisfaction (e.g., Satici and Uysal, 2015), and poor work performance and insomnia (e.g., Brailovskaia et al., 2019, 2020). This widespread usage of the Internet and social media has raised notable concerns about problematic Internet behaviors and related conditions (Young, 2004), which has resulted in the inclusion of a novel diagnostic condition, namely the “Internet Gaming Disorder (IGD)” in the *“as a condition for further study”* section of the *Diagnostic and Statistical Manual of Mental Disorders* (*DSM-5*; American Psychiatric Association, 2013). Technological addictions (e.g., the Internet, cell phones, and SNS) are generally viewed as a type of behavioral addiction characterized by core features of addictions, i.e., salience, withdrawal, and euphoria (e.g., Griffiths, 1996; Elphinston and Noller, 2011). Concerning SNS, high levels of SNS use are characterized by an excessive attachment to social media, which interferes with daily and relationship functioning. Pathological SNS use may also include withdrawal (distress related to an inability to access SNS), relapse and reinstatement (unyielding efforts to reduce SNS), and euphoria (feeling connected to others when using SNS; Elphinston and Noller, 2011). Therefore, the development of reliable measures to assess online social behavior, the nature of social media use, and its potential implications is of utmost importance (e.g., Elhami Athar and Azamian Jazi, 2022).

In this regard, Elphinston and Noller (2011) developed the Facebook Intrusion Questionnaire (FIQ) based on Brown’s (Brown, 1997) behavioral addiction components and the mobile phone involvement questionnaire by Walsh et al. (2010). FIQ includes eight item assessing behavioral addiction components among Facebook users. Items measure the link between the tendency toward Facebook involvement and eight aspects of behavioral addiction, including cognitive salience, behavioral salience, euphoria, interpersonal conflict, conflict with other activities, withdrawal, relapse/reinstatement, and loss of control. The original factor structure analysis yielded a one-factor model structure, which enjoyed adequate model fit indices and internal consistency (*α* = 0.85). This uni-factor model also demonstrated the expected association with relationship dissatisfaction. To our knowledge, only one study has examined the psychometrics of the FIQ, which was performed with 567 Spanish adults (Bendayan and Blanca Mena, 2019). In this sole study, the authors performed exploratory and confirmatory factor analysis and provided support for the originally proposed one-factor model of the FIQ. In addition, the FIQ demonstrated good internal consistency and demonstrated the expected associations with self-control, time spent using social networking sites, problematic mobile phone use, internet addiction, phubbing, fear of missing out, and depression (Bendayan and Blanca Mena, 2019). Moreover, the study on the psychometric properties of the FIQ is in earlier steps and more studies are needed to examine the psychometrics of FIQ within different cultures (e.g., Eastern cultures). Additionally, it is yet to be studied if the proposed uni-factor model of the FIQ is invariant across gender groups.

## This study

As mentioned above, to our knowledge, only one study has examined the psychometrics of the FIQ, which was performed with a community sample from Spain (Bendayan and Blanca Mena, 2019). While the FIQ yielded adequate psychometric properties in this work, the study was performed in a Western country, making it uncertain to what extent findings can be generalized to Eastern societies and whether the measure holds the same factorial structure in various cultures. Also, no study has yet examined the measurement invariance (MI) of the FIQ score among gender groups. The establishment of MI provides evidence of a consistent underlying structure across groups, enabling group means to be compared (Han et al., 2019).

This study was conducted as an attempt to address the above-addressed limitations that hallmarked prior work on FIQ. We first conducted a CFA to examine the originally proposed uni-factor model of the FIQ. Given the cultural differences between Eastern and Western countries (For a review, see Ebrahimi et al., 2021,^2022^; Elhami Athar et al., 2022a,b), there might need for some modifications for this model to yield adequate fit (e.g., Elhami Athar and Azamian Jazi, 2022). Nonetheless, we expected the FIQ score to yield at least acceptable internal consistency scores based on Chronbach’s alpha coefficient. Finally, the associations between the FIQ and the theoretically related variables were examined to explore the validity of the FIQ scores. In this regard, we expected the FIQ yield significant positive associations with external correlates of interests, including time spent using social networking sites, problematic mobile phone use (i.e., addiction to social media based on the cell phone), and depression, and significant negative associations with self-esteem and self-control (e.g., Bendayan and Blanca Mena, 2019).

Of note, since Iranians use various SNS applications such as Instagram and Telegram more than Facebook, we modified the Facebook Intrusion Scale items to assess the intrusion of all SNS applications. To this end, we substituted the word “Facebook” with “social media” in all FIQ items. An example item is, *“I often think about social media when I am not using it.”* Thus, from hereon, we changed the label of the measure from “FIQ” to “Social Media Intrusion Questionnaire (SMIQ).”

## Method

### Participants and procedure

A commonly used rule of thumb for calculating sample size in CFA analysis is to consider 10 to 20 samples per item in a questionnaire (e.g., Jackson, 2003). However, as we aimed to examine the Measurement Invariance of the FIQ across gender groups, we recruited a significantly larger sample. Participants included 374 individuals (aged 18 to 48; M age = 25.91; *SD* = 5.81; 69.80% females) from the community sample in Tehran who were recruited online from December 2021 to April 2022. More specifically, participants were contacted through a secured online platform and were informed about the aims and the voluntary and confidential character of the study; they then provided online informed consent. Participants completed the questionnaires online in a specified order using a secured online platform at a time and location of their convenience.^1^ The participants reported that they used Instagram (338; 90.3%), Telegram (333; 88.8%), WhatsApp (361; 96.3%), YouTube (124; 33.1%), Twitter (56; 14.9%), Facebook (27; 7.2%), LinkedIn (32; 8.5%), and other SNSs (33; 8.8%). This study was first reviewed and approved by the ethics committee of the Iran University of Medical Sciences (code number = IR.IUMS.REC.1400.1189).

### Measures

#### Facebook intrusion questionnaire

The FIQ (Elphinston and Noller, 2011) is an eight-item scale developed to measure the level of Facebook addiction (behavioral addiction) in Facebook users. The eight items assess the link between the tendency toward Facebook involvement and eight aspects of behavioral addiction, including cognitive salience, behavioral salience, euphoria, interpersonal conflict, conflict with other activities, withdrawal, relapse/reinstatement, and loss of control. Items are rated on a 7-point Likert scale ranging from 1 (*strongly disagree*) to 7 (*strongly agree*), which are summed to indicate the level of addiction, with higher scores representing higher Facebook addiction.

##### Persian FIQ

The English version of the FIQ was translated into Persian by the first author (a native Persian speaker and fluent in English) and was back-translated into English by an independent bilingual speaker. Next, the back-translated English version was reviewed by the authors, and a few modifications were made to ensure the clarity of the translations. As explained above, we modified the FIQ items to cover the intrusion of all SNSs by substituting the word “Facebook” with “social media” in all items (An example item: *“I often think about social media when I am not using it.”*) Accordingly, we labeled the measure as Social Media Intrusion Questionnaire (SMIQ).

### Time spent using social media

To determine the time spent using social media, participants were asked: *“Approximately, how long do you spend each day on social media?”* The response options were: Less than 1 h, 1–3 h, 3–5 h, 5–8 h, and more than 8 h.

### The 13-item beck depression inventory

BDI-13 (Beck and Beamesderfer, 1974) was developed to measure the severity of the level of depression over the past week *via* 13 items using statements scored from 0 to 3. The BDI is a well-established questionnaire used to screen for depression, which has been validated for use in non-psychiatric patients, including students (Beck et al., 1988). The Persian version of the BDI-13 demonstrated acceptable psychometric properties (Dadfar and Kalibatseva, 2016). In the present study, the alpha for the BDI-13 was 0.88.

### The mobile-based social networking addiction scale

This measure was developed to assess the levels of addiction to social media based on cell-phone (Khajeahmadi et al., 2017). It consists of 23 items loading on four dimensions, including individual performance, time management, self-control, and social relations. The items are scored using a 5-point Likert type scale ranging from 1 (*strongly disagree*) to 5 (*strongly agree*). Higher scores indicate a higher level of addiction to social media. In the original study with the Iranian sample, the measure yielded acceptable internal consistency. In the present study, the alpha for the total score of this measure was 0.91.

### The Rosenberg self-esteem scale

The Rosenberg self-esteem scale (RSES; Rosenberg, 1965) is the most widely used measure of self-esteem for research purposes. It includes 10 items rating on a Likert scale with items ranging from 1 (*strongly disagree*) to 4 (*strongly agree*). Total scores range from 10 to 40, with higher scores representing lower self-esteem. The scale measures state self-esteem by asking the respondents to reflect on their current feelings. Five of the items have positively worded statements, and five have negatively worded ones. The Persian version of the RSES yielded adequate psychometric properties (Mohammadi, 2005). In the present study, the RSES yielded an alpha of 0.82.

### The brief self-control scale

The BSCS (Tangney et al., 2004) is a 13-item version of the longer Self Control Scale and is designed to focus on the behavioral aspects of self-control, like breaking bad habits or persevering through a task. Items of the BSCS are rated on a 5-point Likert type scale ranging from 1 (*not at all like me*) to 5 (*very much like me*), with higher scores indicating higher levels of self-control. The Persian version of the BSCS yielded acceptable psychometric properties in Iran (Sagar, 2021). In the current study, the alpha for the BSCS was 0.70.

### Data analyses

First, using the JASP free software (JASP Team, 2021), we first conducted a confirmatory factor analysis (CFA) with Diagonally Weighted Least Squares (DWLS) estimator, which is appropriate for ordinal data (Flora and Curran, 2004). We preferred to perform CFA first because when prior research has established the factor structure of a measure, the statistical method used in the later construct validation studies should be confirmatory factor analysis rather than exploratory factor analysis (EFA; Brown, 2015; Taheri et al., 2021). To evaluate model fit, we considered the Tucker–Lewis index (TLI), the comparative fit index (CFI), and the root mean square error of approximation (RMSEA). We considered RMSEA scores below 0.05 to indicate a good fit and scores between 0.05 and 0.08 to indicate acceptable fit. A TLI and CFI score of 0.95 or above indicates excellent fit, and scores of 0.90 or more indicate a good fit (Bentler, 1990; Hu and Bentler, 1999). The proposed uni-factor model of the SMIQ was specified with the eight items as observed variables loading on a latent variable of SMIQ. Next, using the confirmed factorial structure model, we performed measurement invariance (MI) tests across gender groups based on the sequential strategy suggested by Meredith and Teresi (2006). Accordingly, tested the selected model separately for males and females. Also, three levels of MI (i.e., configural, metric, and scalar) were tested to examine whether the factor structure, factor loadings, and item intercepts, respectively, were invariant across groups. Change in CFI (∆CFI) was used as an indicator for testing MI which is independent of model parameters and sample size. According to Cheung and Rensvold (2002), a value of ∆CFI smaller than or equal to 0.01 supports the presence of MI across groups. If the results of the MI test indicate that mean comparisons could be made across gender groups, we will compare the FIQ score across gender groups. We also calculated the internal consistency of the FIQ score with Cronbach’s alpha (*α*; Cronbach, 1951), defined as low-to-marginal (≤0.59), marginal (0.60 to 0.69), acceptable (0.70 to 0.79), good (0.80 to 0.89), and excellent (≥0.90; Cheung and Rensvold, 2002). Finally, zero-order correlations between the FQI score and the external criterion variables were computed. Correlation coefficients were interpreted as ≤0.30 = small; 0.30–0.50 = medium; and ≥0.50 = strong effect sizes (Cohen, 2013). All analyses were conducted in SPSS 20 unless otherwise specified. A *p*-value of <0.05 was considered as the indicator of statistical significance.

## Results

### Confirmatory factor analyses and measurement invariance tests

**Figure 1:**
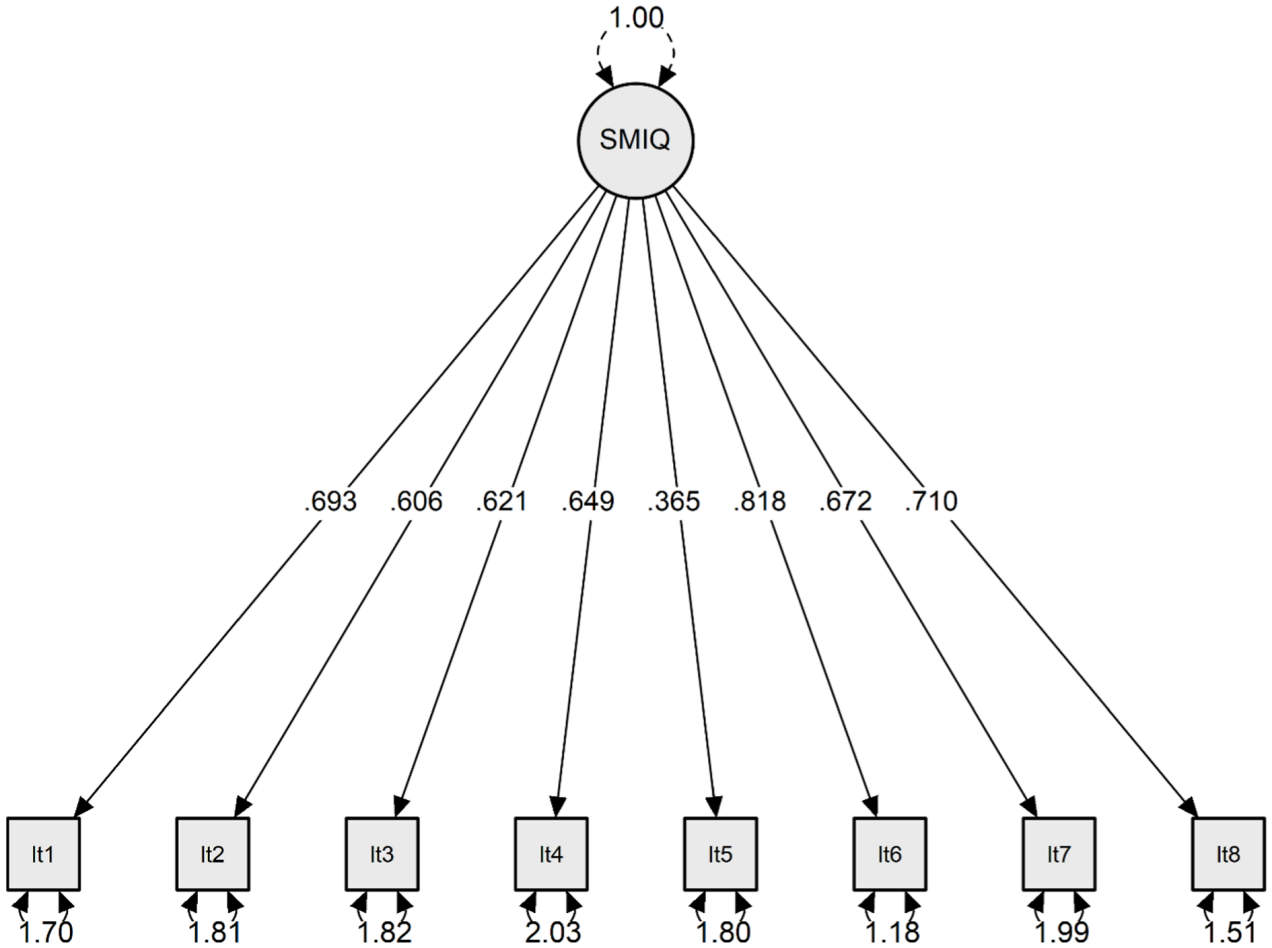
Standardized model parameters for the confirmatory factor analysis: the uni-mode of the SMIQ. SMIQ = social media intrusion questionnaire.

Confirmatory factor analysis results indicated that the originally proposed one-factor model of the SMIQ yielded excellent (RMSEA = 0.022; CFI = 0.998; TLI = 0.997). The standardized loadings of the FIQ items can be retrieved from Figure 1 and Table 1. We further performed MI tests across gender groups. The one-factor model with eight items was first tested across gender groups separately, which yielded excellent model fit for males (RMSEA = 0.001; CFI = 0.998; TLI = 0.997) and females (RMSEA = 0.001; CFI = 0.999; TLI = 0.998). Then, configural, metric, and scalar invariance were examined in sequence for gender groups, and the results showed that model fit indices were in the excellent range for configural (RMSEA = 0.001, CFI = 0.999, TLI = 0.999), metric (RMSEA = 0.032; CFI = 0.995; TLI = 0.994) and scalar (RMSEA = 0.027, CFI = 0.996, TLI = 0.996) invariances.

**Table 1:** Standardized loadings of the SMIQ items (*n* = 374).

Item	Item abbreviation	Std. estimation
Item 1	I often think about social media when I am not using it.	0.693
Item 2	I often use social media for no particular reason.	0.606
Item 3	Arguments have arisen with others because of my social media use.	0.621
Item 4	I interrupt whatever else I am doing when I feel the need to access social media.	0.649
Item 5	I feel connected to others when I use social media.	0.365
Item 6	I lose track of how much I am using social media.	0.818
Item 7	The thought of not being able to access social media makes me feel distressed.	0.672
Item 8	I have been unable to reduce my social media use.	0.710

All loadings were significant at *p* < 0.001.

### Internal consistency of the SMIQ and descriptive information

Descriptive statistics for the study variables are presented in Table 2. As the uni-factor model of the SMIQ yielded excellent MI results, we compared the mean SMIQ scores across gender groups; however, no significant differences were found in the SMIQ score between males and females. In terms of internal consistency, the alpha (*α*) for the SMIQ score was 0.85, which is in the good range.

**Table 2:** Mean and standard deviation scores for the SMIQ.

Measures	Total sample (*n* = 374)	Gender			
		Males (*n* = 113)	Females (*n* = 261)	Group Comparison^a^	
	M (*SD*)	M (*SD*)	M (*SD*)	*p*	*d*
SMIQ	31.68 (9.87)	30.53 (10.31)	32.19 (9.65)	0.13	0.17
Cell phone based addiction to social media	56.63 (16.58)	56.44 (16.99)	56.71 (16.44)	-	-
Depression	4.97 (5.55)	4.62 (5.96)	5.11 (5.36)	-	-
Self-control	46.93 (5.16)	46.73 (5.28)	47.01 (5.11)	-	-
Self-esteem	5.75 (4.85)	6.61 (4.28)	5.37 (5.04)	-	-

SMIQ = social media intrusion questionnaire; M = mean; SD = standard deviation; *d* = Cohen’s *d*.

a Gender differences tests were examined only for the SMIQ score because no MI analysis was performed for other variables in this study.

### Convergent and divergent validity of the SMIQ

As shown in Table 3, the results indicated that the SMIQ yielded significant positive associations with cell phone based addiction to social media, time spent using social media, and depression scores (*r*’s = 0.26 to 0.73). The SMIQ also yielded significant negative associations with self-esteem and self-control scores (*r*’s = −0.28 and − 0.35, respectively).

**Table 3:** Pearson correlation between SMIQ and external correlates of interests (*n* = 374).

Measure	Cell phone based addiction to social media	Depression	Self-control	Self-esteem	Time spent using social media
SMIQ	0.731^**^	0.261^**^	−0.352^**^	−0.281^**^	0.485^**^

SMIQ = social media intrusion questionnaire.

** *p* < 0.001.

## Discussion

This study aimed to examine the psychometric properties of the Persian version of the SMIQ (original FIQ) among an Iranian community sample. While FIQ was originally designed to assess addiction to Facebook, we modified the Facebook Intrusion Scale to cover all SNSs by substituting the word “Facebook” with “social media” in all eight items and labeled the measure as SMIQ. First, we conducted a CFA to examine the originally proposed one-factor model of the measure, and the results indicated that this model reached excellent model fit, which is in line with previous studies (Elphinston and Noller, 2011; Bendayan and Blanca Mena, 2019). Furthermore, this study was the first to examine the measurement invariance (MI) of the SMIQ score across gender groups, with the results indicating that the SMIQ is invariant across gender groups, making it possible to compare the scores across gender groups. However, no significant difference was found across males and females in the SMIQ score in this study. The results also indicated that when relying on Cronbach’s alpha (*α*), the internal consistency of the SMIQ score was in the good range consistent with prior research (Elphinston and Noller, 2011; Bendayan and Blanca Mena, 2019).

We also examined the correlations between SMIQ and external criterion measures to examine the convergent and discriminant validity of the Persian SMIQ score. In line with previous studies, it is expected that individuals with higher SMIQ scores have higher levels of addiction to social media and spend more time on SNS. In line with this hypothesis, the results showed that the SMIQ was significantly and positively associated with scores of cell phone based addiction to social media and time spent using social media (Elphinston and Noller, 2011; Bendayan and Blanca Mena, 2019; Elhami Athar and Azamian Jazi, 2022). Likewise, prior works suggested that increased SNS usage may lead to negative online experiences and fewer in-person interactions. It could also result in the development of attachment to social media, which could make individuals susceptible to negative social interactions and feedback, and, subsequently, higher risks for depression (e.g., Baek et al., 2013; Shensa et al., 2018). In this vein, our results indicated that the SMIQ had significant positive associations with depression scores. All these results support the convergent validity of the SMIQ score. In addition, it has been shown that more frequent SNS use is linked with lower self-esteem due to increased exposure to high-level social comparisons (e.g., Vogel et al., 2014). In line with this finding, our results showed that higher SMIQ scores were negatively and significantly associated with lower self-esteem scores. Finally, as SNS is constantly available and associated with rewards mechanisms, it may challenge individuals’ self-control abilities in terms of controlling the frequency and amount of SNS usage (Turel and Qahri-Saremi, 2016). Thus, as expected based on this finding, the SMIQ yielded a significant negative correlation with self-control abilities. Altogether, these results provided support for the convergent and divergent validity of the SMIQ scores.

Our results should be interpreted considering some limitations. First, we examined the convergent/discriminant validity of the SMIQ using the self-report measure, so it is possible that the correlations between the measures may partly be explained by shared method variance. Second, our study sample included participants from the community, so the results from this study should not be generalized to other samples.

Despite these limitations, our results indicated that the SMIQ is a valid and reliable measure of addiction to social media. Our study would encourage researchers to replicate our results in various cultures and countries to bolster the literature on the psychometrics of the SMIQ (original FIQ).

## Data availability statement

The raw data supporting the conclusions of this article will be made available by the authors, without undue reservation.

## Ethics statement

The studies involving human participants were reviewed and approved by Research Deputy of the Iran University of Medical Sciences. The patients/participants provided their written informed consent to participate in this study.

## Author contributions

ZA gathered the data, performed data analyses, and prepared the manuscript. AA, ARF, and HF supervised the study and reviewed and revised the manuscript. All authors contributed to the article and approved the submitted version.

## Conflict of interest

The authors declare that the research was conducted in the absence of any commercial or financial relationships that could be construed as a potential conflict of interest.

## Publisher’s note

All claims expressed in this article are solely those of the authors and do not necessarily represent those of their affiliated organizations, or those of the publisher, the editors and the reviewers. Any product that may be evaluated in this article, or claim that may be made by its manufacturer, is not guaranteed or endorsed by the publisher.

## Footnotes

^1^Participants were required to respond to all items in the online platform. Therefore, there was no missing data in the current study.
